# Supplementary material for: Evolutionary action of mutations reveals antimicrobial resistance genes in Escherichia coli
Source: Nat Commun. 2022 Jun 9;13:3189. doi: 10.1038/s41467-022-30889-1 (PMC9184624; doi:10.1038/s41467-022-30889-1)
Supplement: Supplementary file 1 — Supporting information [file 41467_2022_30889_MOESM1_ESM.pdf]

Supporting information for

Evolutionary action of mutations reveals antimicrobial resistance genes in *Escherichia coli*

David C. Marciano †,*, Chen Wang†, Teng-Kuei Hsu, Thomas Bourquard, Benu Atri, Ralf B. Nehring, Nicholas S. Abel, Panagiotis Katsonis, Elizabeth A. Bowling, Taylor J. Chen, Pamela D. Lurie, Susan M. Rosenberg, Christophe Herman and Olivier Lichtarge*

*To whom correspondence should be addressed.

†The authors wish it to be known that, in their opinion, the first two authors should be regarded as Joint First Authors

Contact: lichtarge@bcm.edu; david.marciano@bcm.edu

This section includes:

Supplementary Figures 1 to 13, pages 2-14

Supplementary Tables 1 to 4, pages 15-18

Additional References, page 19

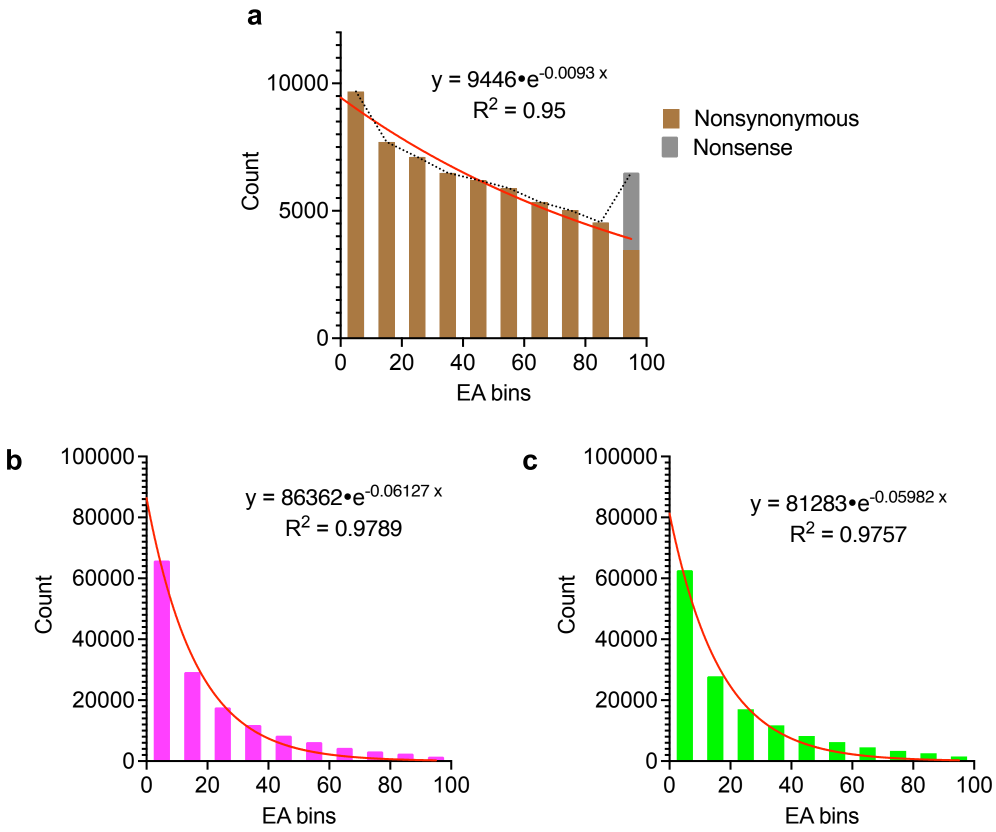

Supplementary Figure 1, Related to Figure 1. The EA distribution of background mutations. The EA scores are grouped into 10 equally sized bins. The mutation count for the nonsynonymous mutations and the mid-point of each bin was fitted with an exponential decay model (red line) with high goodness of fit. (**a**) In silico generated background EA distributions for lab evolved MG1655. Mutations were simulated by randomly generating nucleotide substitutions in MG1655 genome. As EA theory has not fully developed its prediction of the impact of nonsense mutations, we currently assign those mutations with EA = 100. The combination of simulated nonsynonymous and nonsense mutations were used as the background distribution (dashed line) for our EA integral analysis for our lab evolved samples. (**b, c**) Background EA distributions for ciprofloxacin (**b**) and colistin (**c**) clinical/environmental *E. coli* strains. Mutations were obtained by comparing the protein sequences from the sensitive strains with the *E. coli* MG1655 reference genome (NC000913). Since protein sequences were used, only nonsynonymous mutations are reported. Identical mutations observed in different strains are only counted once. Source data are provided as a Source Data file.

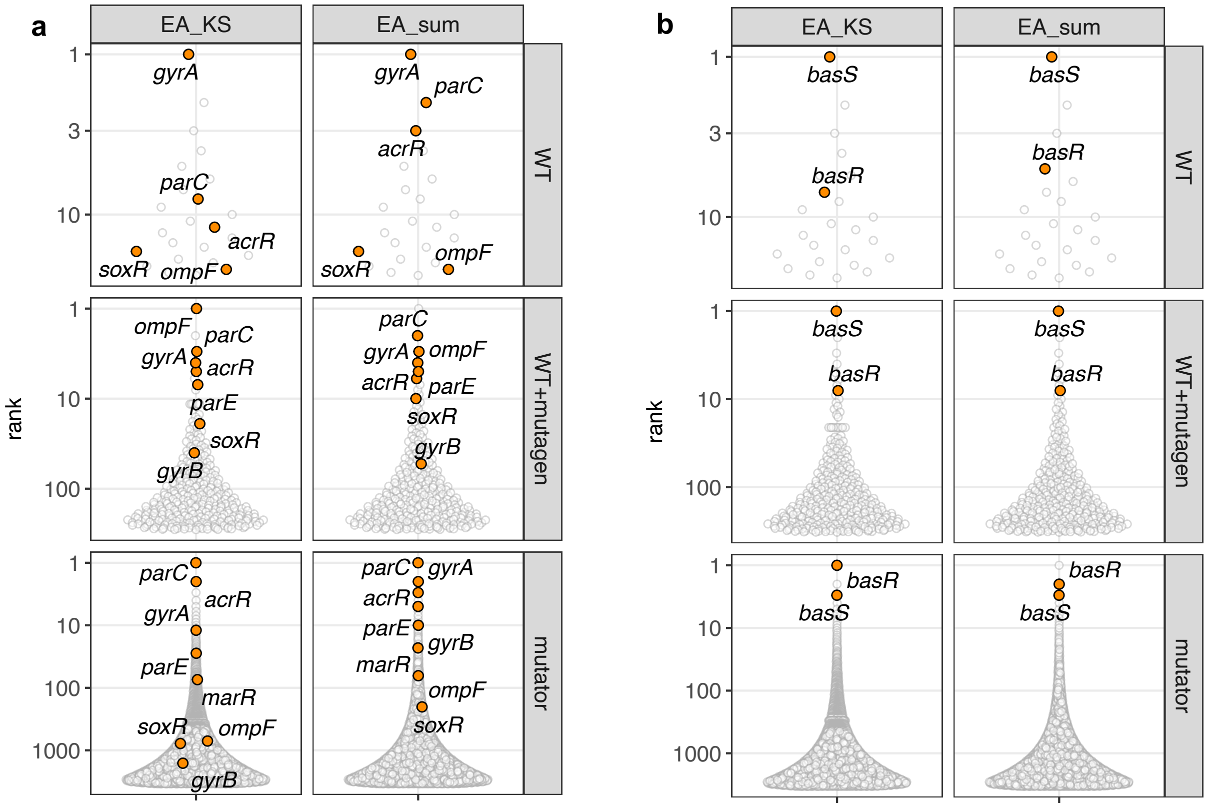

Supplementary Figure 2, Related to Figure 2. EA integration recovers most known drivers in each mutation/selection condition. The individual rankings are plotted for ciprofloxacin or colistin datasets using either a KS test (EA-KS) or the mutation rate adjusted summation of EA scores (EA-sum). Genes previously shown to contribute towards either ciprofloxacin resistance (**a**) or colistin resistance (**b**) are shown in orange.
Source data are provided as a Source Data file.

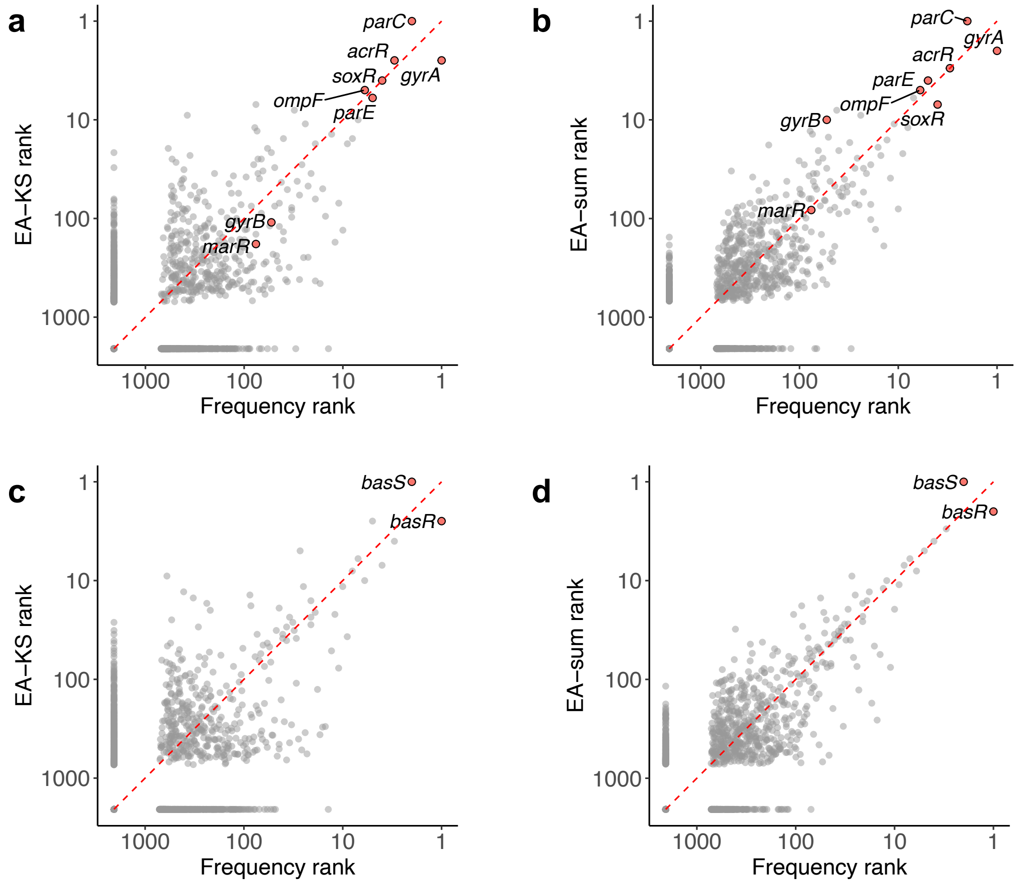

e

Jaccard similarity coefficient of top 20 genes predicted by different methods		
	EA-KS vs Freq	EA-sum vs Freq
ciprofloxacin	0.379	0.39
colistin	0.3	0.56

Supplementary Figure 3, Related to Figure 3. Comparisons of EA integration with frequency-based analysis in lab evolved strains. Frequency analysis and EA integration ranks (EA-KS and EA-sum) of the mutated genes in ciprofloxacin resistant (**a, b**) and colistin resistant (**c, d**) lab evolved strains. Known drivers (orange) are highlighted. (**e**) The Jaccard similarity coefficient for the top 20 genes predicted by EA integration methods and frequency-based method. Source data are provided as a Source Data file.

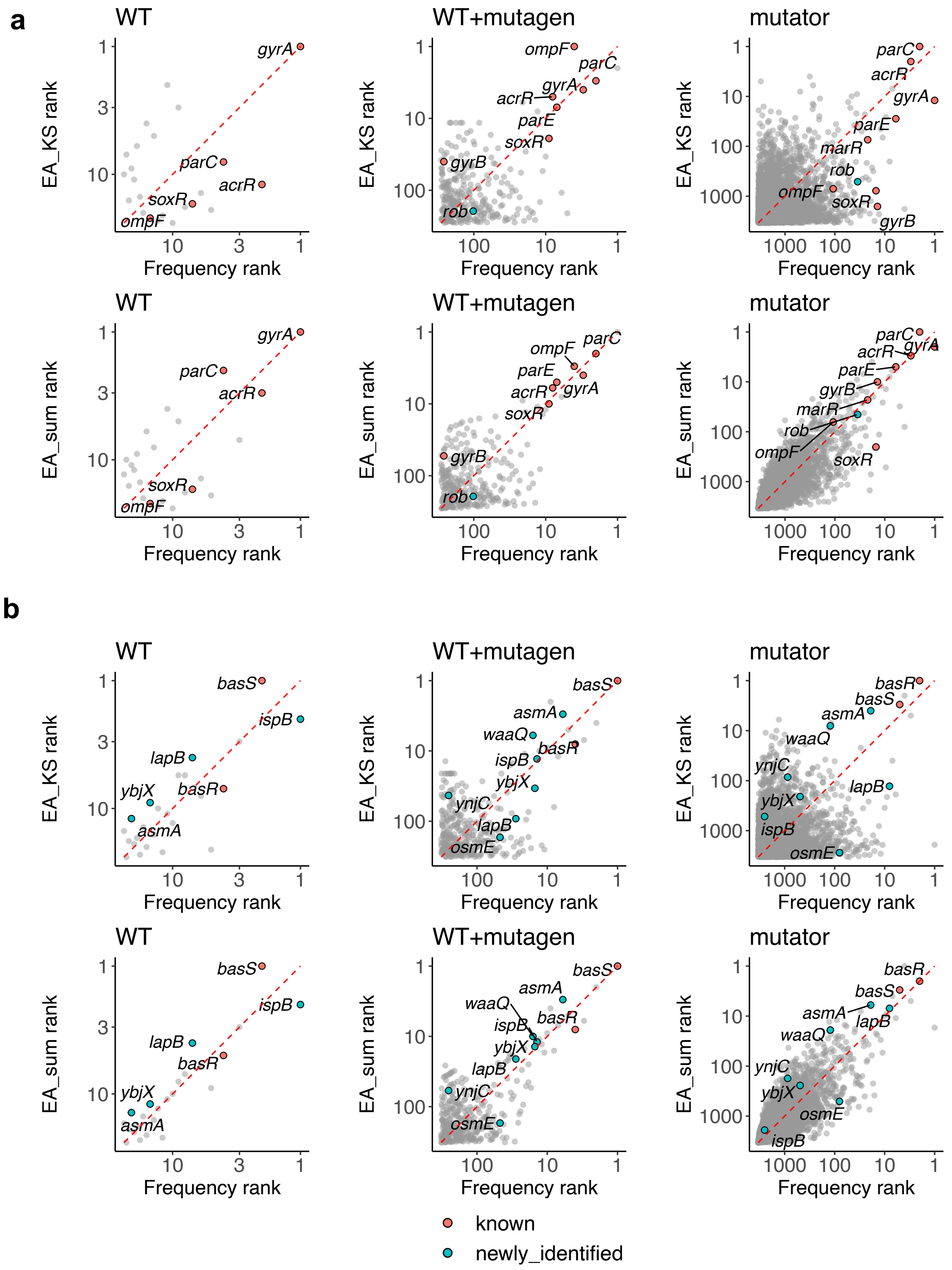

Supplementary Figure 4. Comparisons of EA integration with frequency-based analysis in lab evolved strains under different mutational loads. Frequency analysis and EA integration ranks (EA-KS and EA-sum) of the mutated genes in ciprofloxacin resistant (**a**) and colistin resistant (**b**) lab evolved strains are shown for each mutational load. Known drivers and newly identified drivers are shown as red and blue, respectively. Source data are provided as a Source Data file.

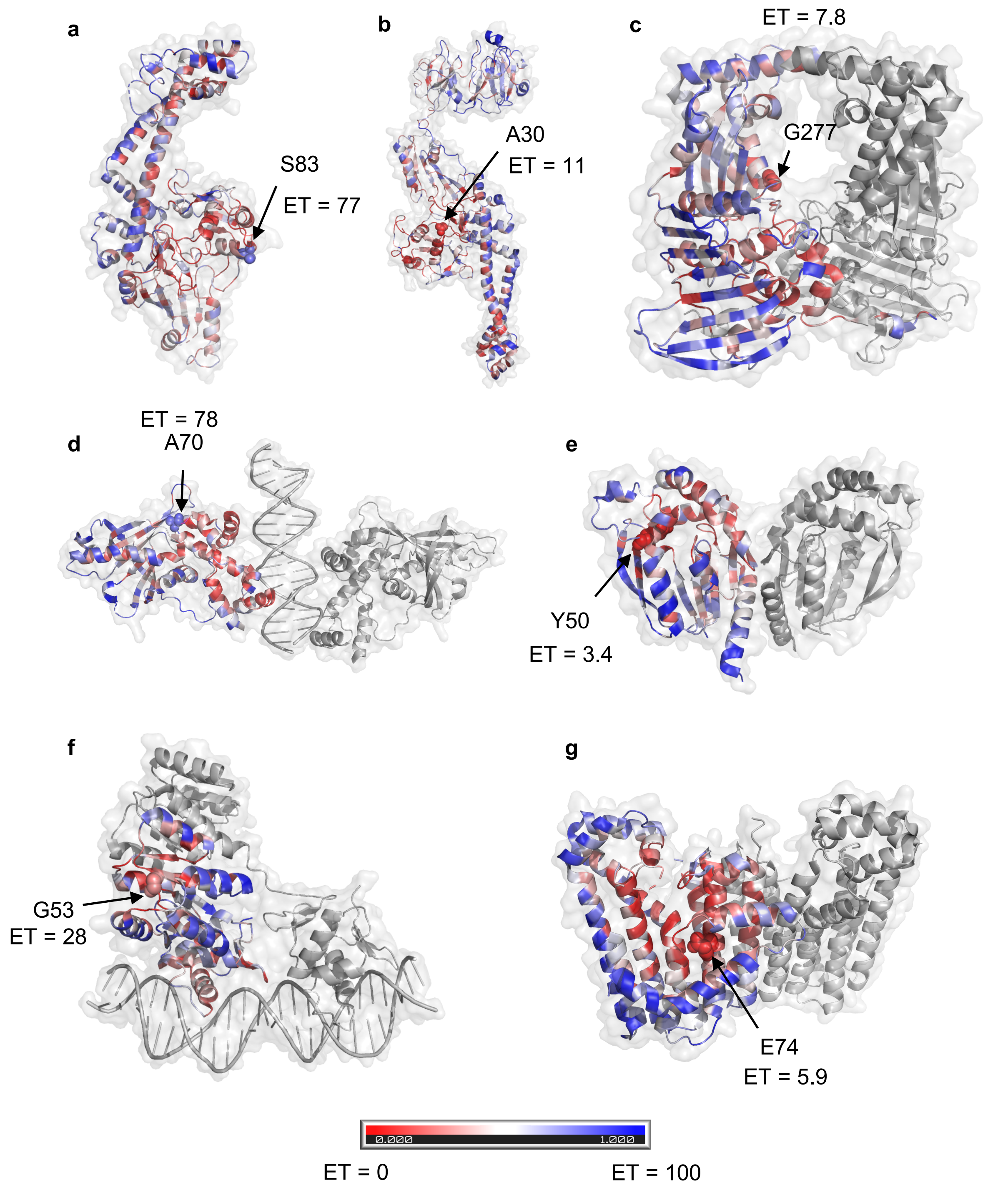

Supplementary Figure 5, Related to Figure 3. ET coverage mapped to the crystal structure of selected genes or their homologs. Only chain A was colored according to ET. A lower (red) ET coverage suggests greater evolutionary importance. (**a**) gyrA (1AB4 [http://doi.org/10.2210/pdb1AB4/pdb]) [1]; (**b**) parC (1ZVU [http://doi.org/10.2210/pdb1ZVU/pdb]) [2]; (**c**) parE (1S16 [http://doi.org/10.2210/pdb1S16/pdb]) [3]; (**d**) rob (1D5Y [http://doi.org/10.2210/pdb1D5Y/pdb]) [4]; (**e**) udk homolog from *Thermus thermophilus* (3W8R [http://doi.org/10.2210/pdb3W8R/pdb]) [5]; (**f**) basR homolog from *Klebsiella pneumoniae* JM45 (4S04 [http://doi.org/10.2210/pdb4S04/pdb]) [6]; (**g**) ispB (3WJK [http://doi.org/10.2210/pdb3WJK/pdb]) [7]

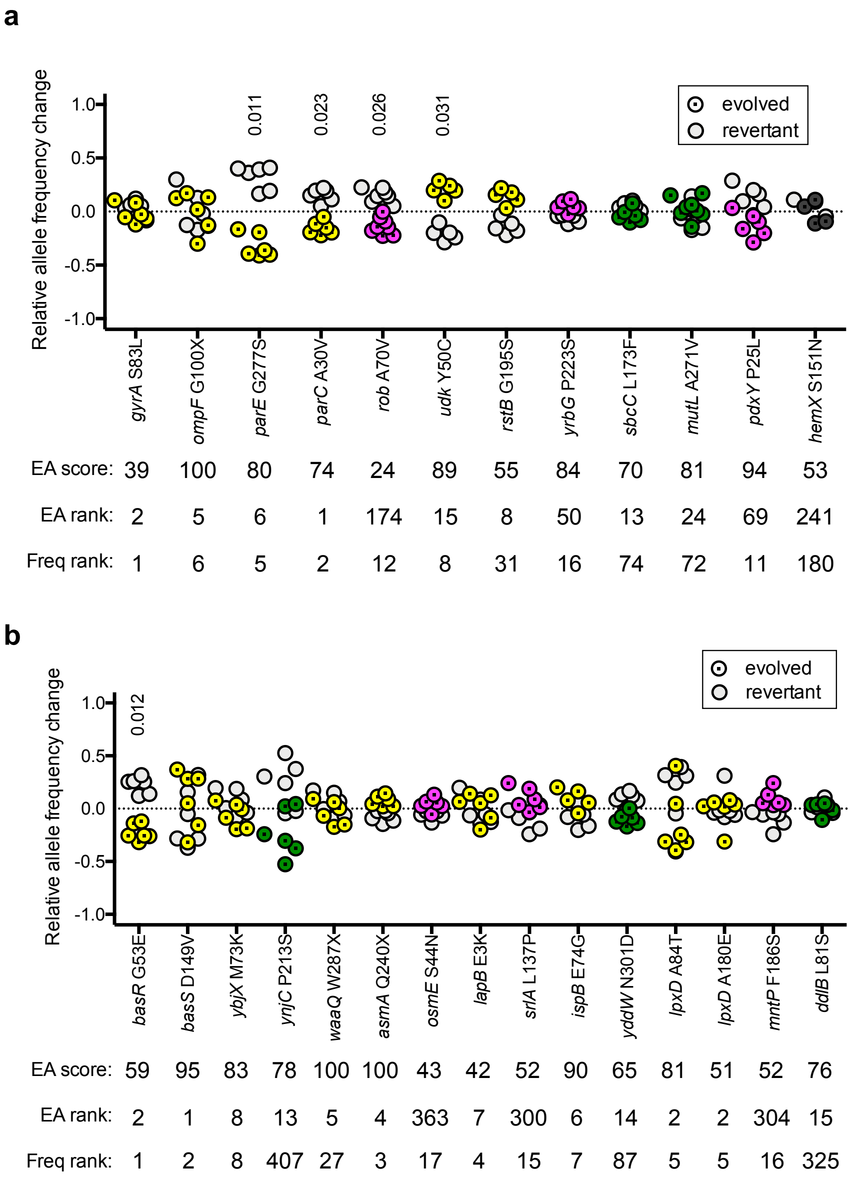

Supplementary Figure 6, Related to Figure 3. Contribution of specific mutations to fitness in the absence of antibiotic. As a negative control, all revertants were competed against the evolved strains in rich media without antibiotics. N=4 independent biological samples for hemX. N=5 independent biological samples for *udk*, *rstB*, and *ispB*. N=8 independent biological samples for *rob* and *mutL*. All other genes were repeated with n=6 independent biological samples. Data points colored yellow if gene is ranked highly both EA integrals and frequency analysis, green if EA specific, and magenta if frequency specific. Isogenic revertants shown in grey. One sample two-sided t-test ($\mu_0 = 0$) with Bonferroni correction was performed for each competition assay (p values <0.05 reported above each dataset). With the exception of *udk* Y50C, all tested mutations either have no measured fitness effect or, display a fitness cost in the absence of antibiotic (*rob* A70V, *parE* G277S, *parC* A30V and *basR* G53E). Source data are provided as a Source Data file.

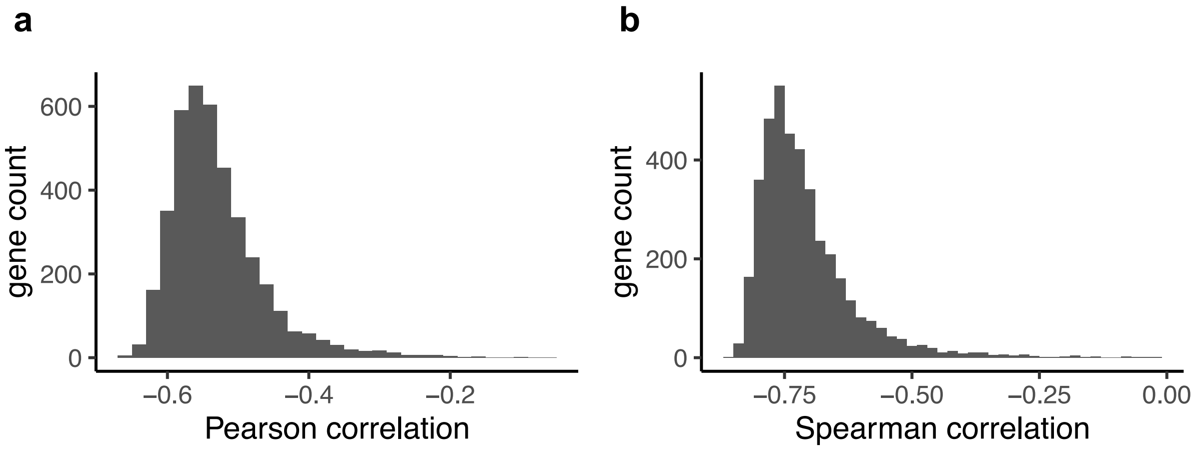

Supplementary Figure 7. EA scores have a good correlation with SIFT scores. Pre calculated SIFT scores for all mutations in MG1655 were downloaded from the mutfunc database (http://mutfunc.com) [8,9]. 4003 genes in MG1655 that have both EA and SIFT scores computed. The Pearson (**a**) and Spearman (**b**) correlations of EA and SIFT scores were calculated for all mutations for each of those genes. Note that mutations with smaller SIFT scores and higher EA are considered more deleterious. Thus, negative correlations between EA and SIFT scores are expected. Source data are provided as a Source Data file.

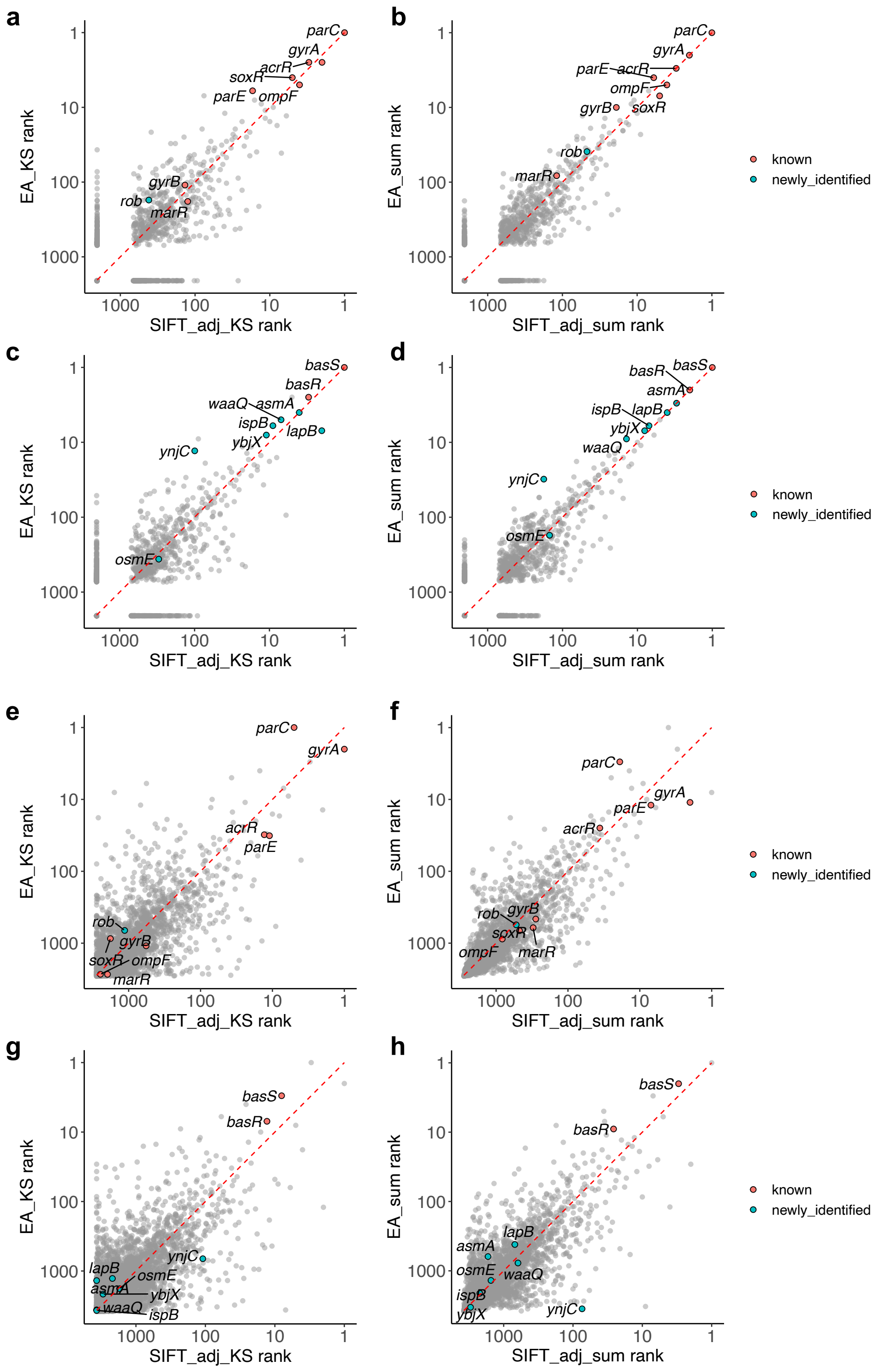

Supplementary Figure 8. EA scores can be substituted with SIFT scores without losing much sensitivity in predicting driver genes. Pre-calculated SIFT scores for *E. coli* MG1655 were obtained from the mutfunc database (http://mutfunc.com) [8,9] and mapped to mutations in the samples. Any mutation that was not annotated by either SIFT or EA were removed. SIFT scores were scale by 100 * (1-SIFT) to the same range and direction as EA (0 and 100 as least and most impactful mutations, respectively). Integration of SIFT_adj scores were done similarly as EA scores. And the gene ranks by EA and SIFT_adj integrations (KS: **a, c, e, g**; sum: **b, d, f, h**) were compared. Known drivers and newly identified drivers were labelled as red and blue, respectively. Ciprofloxacin ALE: **a, b**. Colistin ALE: **c, d**. Ciprofloxacin environmental: **e, f**. Colistin clinical: **g, h**. Source data are provided as a Source Data file.

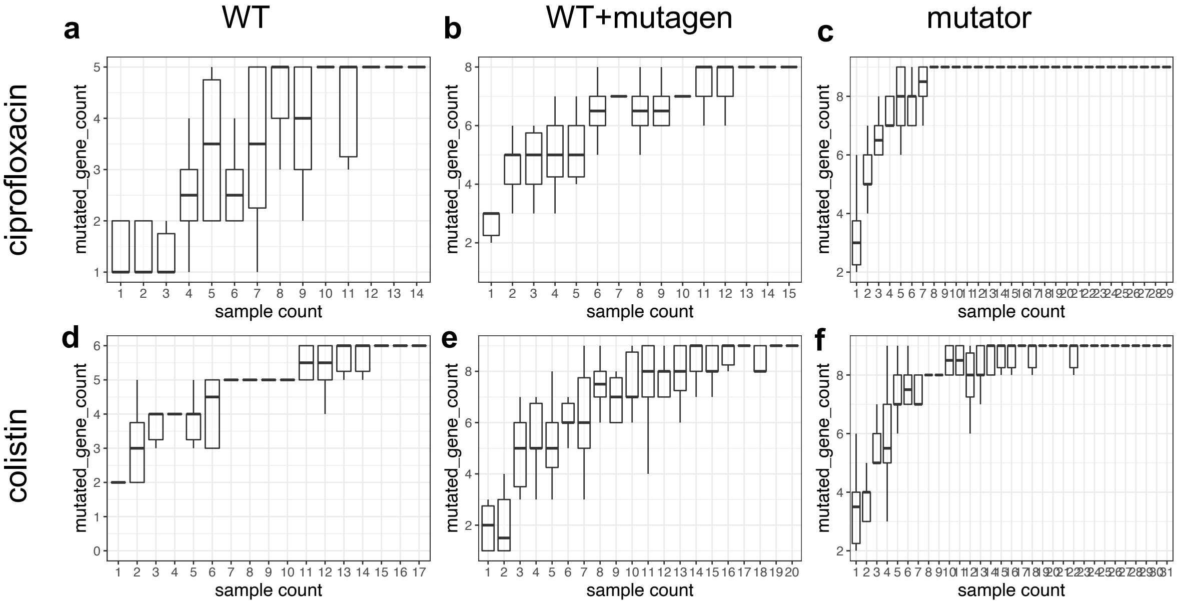

Supplementary Figure 9. Number of drivers genes mutated in the downsampling analyses. Subsamples with different sizes were randomly drawn from the full datasets. The number of phenotypic drivers (ciprofloxacin: *gyrA*, *gyrB*, *marR*, *acrR*, *parC*, *parE*, *soxR*, *ompF* and *rob*; colistin: *basS*, *basR*, *lapB*, *waaQ*, *asmA*, *ybjX*, *ispB*, *ynjC*, and *osmE*) that have at least one mutation in the subsample were determined. Each sample size was repeated 10 times (n=10 random draws from the original samples). Median is shown as the box center. Interquartile range is shown as the bounds of the box. The maxima and minima are shown as whiskers. Source data are provided as a Source Data file.

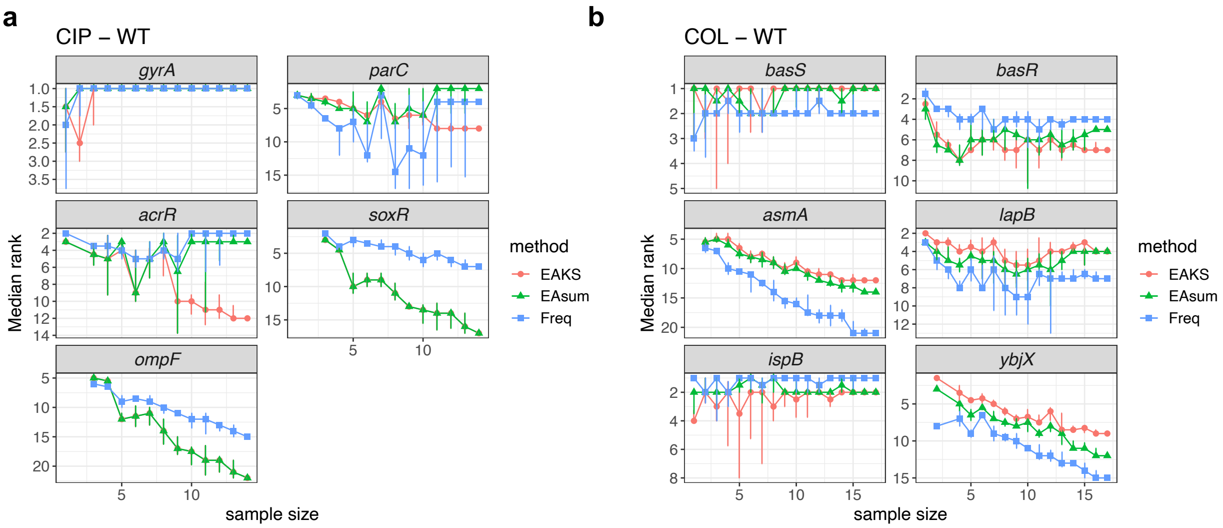

Supplementary Figure 10. Downsampling analyses for the WT samples. The median rank of the top ranked drivers for ciprofloxacin (**a**) and colistin (**b**) in the downsampling analyses for the WT samples are shown. Interquartile range is displayed as error bars from 10 independent random draws at the indicated sample size. Source data are provided as a Source Data file.

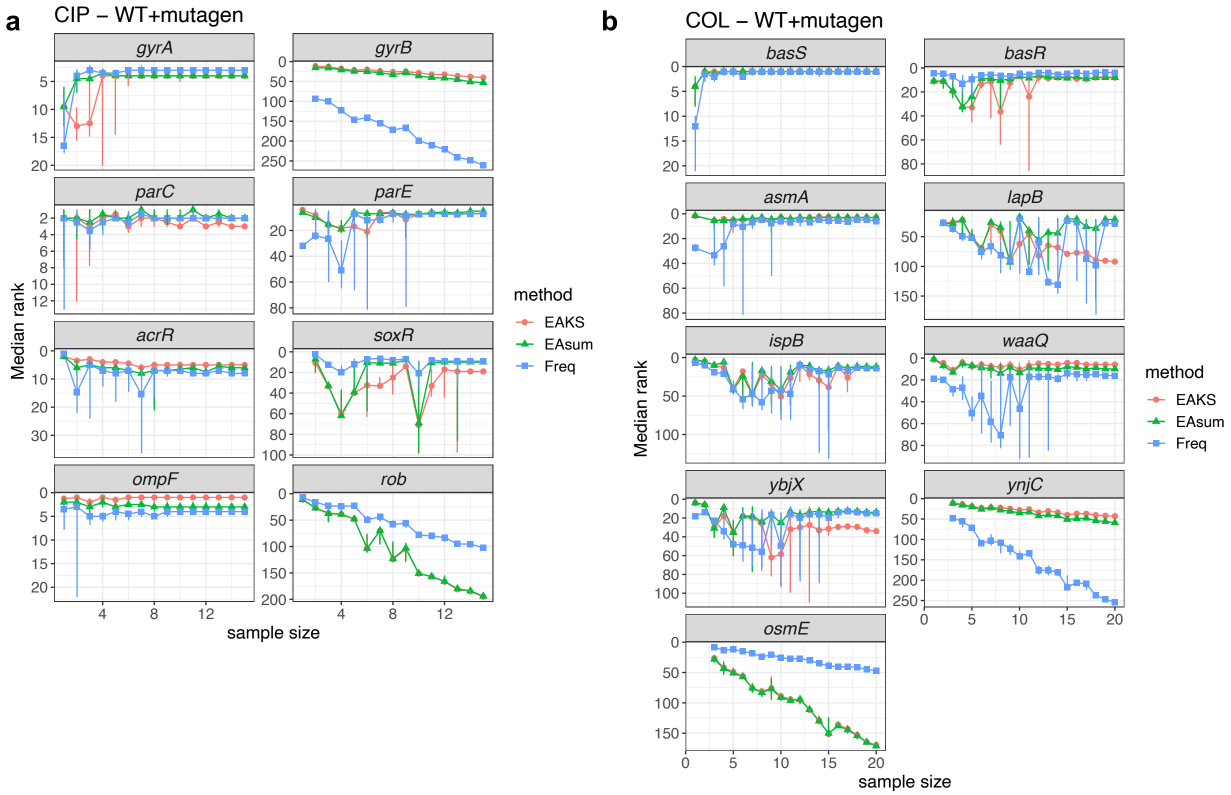

Supplementary Figure 11. Downsampling analyses for the WT+mutagen samples. The median rank of the top ranked drivers for ciprofloxacin (**a**) and colistin (**b**) in the downsampling analyses for the WT+mutagen samples were shown. Interquartile range is displayed as error bars from 10 independent random draws at the indicated sample size. Source data are provided as a Source Data file.

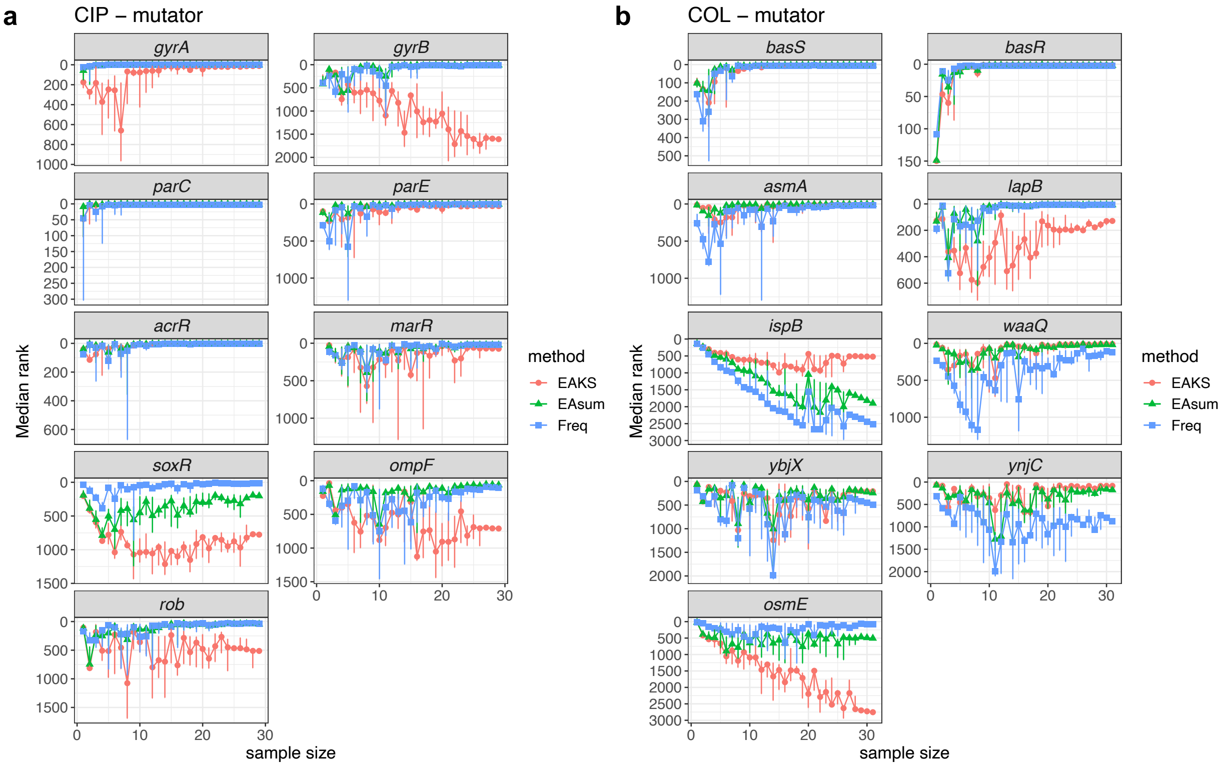

Figure S12. Downsampling analyses for the mutator samples. The median rank of the top ranked drivers for ciprofloxacin (**a**) and colistin (**b**) in the downsampling analyses for the mutator samples were shown. Interquartile range is displayed as error bars from 10 independent random draws at the indicated sample size. Source data are provided as a Source Data file.

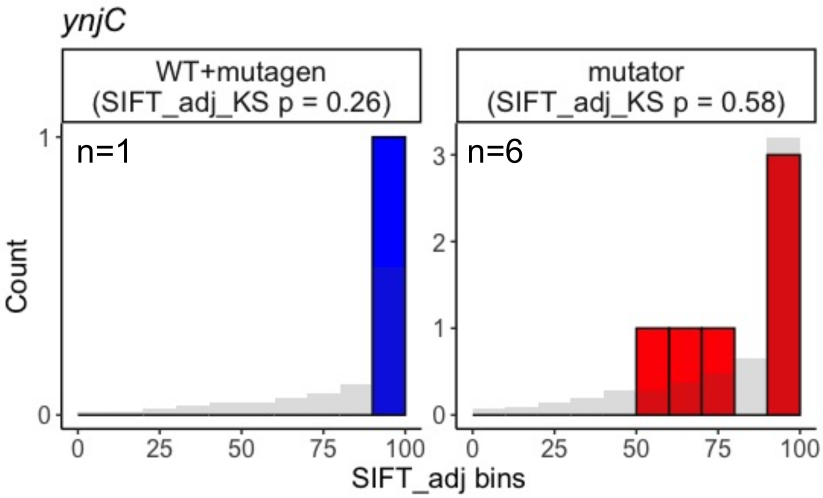

Supplementary Figure 13. Mutations in *ynjC* in the colistin resistance ALE sample are uncommon but impactful. The EA distributions of *ynjC* mutations in colistin ALE samples compared to randomly simulated mutations (gray bars) with results of Kolmogorov-Smirnov (EA-KS) test shown. The number of mutations observed in each mutational conditions are label in the figure. *YnjC* was not commonly mutated in the samples, but those mutations were predicted to be impactful by EA. Source data are provided as a Source Data file.

Supplementary Table 1, Related to Figure 3. EA scores for mutations tested in competition experiments.

Mutation	EA score	Antibiotics
gyrA S83L	39	ciprofloxacin
ompF G100X	100	ciprofloxacin
parE G277S	80	ciprofloxacin
parC A30V	74	ciprofloxacin
udk Y50C	89	ciprofloxacin
rstB G195S	55	ciprofloxacin
yrbG P223S	84	ciprofloxacin
sbcC L173F	70	ciprofloxacin
hemX S151N	53	ciprofloxacin
mutL A271V	81	ciprofloxacin
pdxY P25L	94	ciprofloxacin
basR G53E	59	colistin
ybjX M73K	83	colistin
basS D149V	95	colistin
ynjC P323L	78	colistin
waaQ W287X	100	colistin
asmA Q240X	100	colistin
lapB E3K	42	colistin
srlA L137P	52	colistin
ispB E74G	90	colistin
yddW N301D	65	colistin
lpxD A84T	81	colistin
lpxD A180E	51	colistin
mntP F186S	52	colistin
ddlB L81S	76	colistin

Supplementary Table 2, Related to Figure 3. Ciprofloxacin MIC levels for selected strains. Each strain was tested at least 3 times. If all the measurements agree, a single MIC value is reported, otherwise multiple MICs and the frequencies they appear are reported. Star (*) indicates that strain was obtained from an ALE experiment.

Strain	MIC (μg/mL)
DCM292 (MG1655-2AP/ZEB* *gyrA* S83L *ompF* G100X:Y316H *parC* A30V *udk* Y50C *sbcC* L173F)	4.096
DCM282 (DCM292 $gyrA^{+}$)	0.064
DCM279 (DCM292 $ompF^{+}$)	2.048
DCM273 (DCM292 $parC^{+}$)	1.024
DCM285 (DCM292 udk^{+})	2.048
DCM262 (DCM292 $sbcC^{+}$)	4.096
DCM293 (MG1655-2AP/ZEB* *parE* G277S	1.024/2.048 (2/1)
DCM275 (DCM293 $parE^{+}$)	1.024
DCM295 (MG1655-2AP/ZEB* *rob* A70V)	8.192
DCM267 (DCM295 rob^{+})	4.096
DCM291 (MG1655-2AP/ZEB* *rstB* G195S)	32.768
DCM260 (DCM291 $rstB^{+}$)	32.768
DCM290 (MG1655-2AP/ZEB* *yrbG* P223S)	4.096
DCM258 (DCM290 $yrbG^{+}$)	4.096
DCM294 (MG1655-2AP/ZEB* *mutL* A271V)	2.048
DCM265 (DCM294 $mutL^{+}$)	1.094/2.096(1/2)
DCM296 (MG1655-2AP/ZEB* *pdxY* P25L)	4.096/8.192 (3/1)
DCM284 (DCM296 $pdxY^{+}$)	4.096/8.192 (2/1)
DCM289 (MG1655-2AP/ZEB* *hemX* S151N)	2.048/4.096 (1/2)
DCM256 (DCM289 $hemX^{+}$)	2.048/4.096 (2/1)
MG1655	0.064
DCM309 (MG1655 *gyrA* S83L)	0.512/1.024 (2/1)
DCM308 (MG1655 *parE* G277S)	0.032/0.064 (1/2)
DCM306 (MG1655 *parC* A30V)	0.032/0.064 (2/2)
CW227 (MG1655 *rob* A70V)	0.128
DCM325 (MG1655 *udk* Y50C)	0.064/0.128 (2/1)
DCM321 (MG1655 *rstB* G195S)	0.064/0.128 (2/1)
DCM319 (MG1655 *yrbG* P223S)	0.064/0.128 (2/1)
DCM323 (MG1655 *sbcC* L173F)	0.064
DCM304 (MG1655 *mutL* A271V)	0.064/0.128 (1/2)
DCM317 (MG1655 *hemX* S151N)	0.064/0.128 (1/2)

Supplementary Table 3, Related to Figure 3. Colistin MIC levels for selected strains. Each strain was tested at least 3 times. If all the measurements agree, a single MIC value is reported, otherwise multiple MICs and the frequencies they appear are reported. Star (*) indicates that strain was obtained from an ALE experiment.

Strain	MIC (μg/mL)
CW120 (MG1655-2AP/ZEB* *basR* G53E, *lapB* E3K)	16/32 (1/2)
CW129 (CW120, *basR*$^{+}$)	<=0.25
CW138 (CW120, *lapB*$^{+}$)	16
CW123 (MG1655-2AP/ZEB* *basR* G53E, *ispB* E74G)	16
CW130 (CW123, *basR*$^{+}$)	<=0.25
CW139 (CW123, *ispB*$^{+}$)	16
CW135 (MG1655* *basS* D149V, *lpxD* A180E)	32
CW142 (CW135, *basS*$^{+}$)	<=0.25
CW144 (CW135, *lpxD*$^{+}$)	32
CW158 (MG1655-2AP/ZEB* *basS* C84R, *lpxD* A84T)	32
CW167 (CW158, *basS*$^{+}$)	<=0.25
CW169 (CW158, *lpxD*$^{+}$)	32
CW126 (MG1655-2AP/ZEB* *basS* C84R, *ybjX* M73K)	64
CW131 (CW126, *basS*$^{+}$)	<=0.25
CW162 (CW126, *ybjX*$^{+}$)	16
CW157 (MG1655-2AP/ZEB* *basS* C84R, *asmA* Q240X)	32
CW165 (CW157, *basS*$^{+}$)	<=0.25
CW166 (CW157, *asmA*$^{+}$)	16/32 (1/2)
CW171 (MG1655-2AP/ZEB* *basS* C84R, *waaQ* W287X)	32
CW176 (CW171, *basS*$^{+}$)	<=0.25
CW177 (CW171, *waaQ*$^{+}$)	16/32 (2/1)
CW181 (MG1655-2AP/ZEB* *basS* L10P, *ynjC* P213S)	16
CW184 (CW181, *basS*$^{+}$)	<=0.25
CW185 (CW181, *ynjC*$^{+}$)	8/16 (1/3)
MG1655	0.0625/0.125 (1/4)
CW103 (MG1655 *basR* G53E)	16
CW105 (MG1655 *basS* C84R)	16
CW223 (MG1655 *osmE* S44N)	0.125/0.25 (2/1)
CW230 (MG1655 *ynjC* P213S)	0.0625/0.125 (1/2)
CW160 (MG1655 *ispB* E74G)	0.0625/0.125/0.25 (2/3/1)
DCM311 (MG1655 *ybjX* M73K)	0.0625/0.125 (2/1)
CW152 (MG1655 *lapB* E3K)	0.0625/0.125 (1/2)

Supplementary Table 4, Related to Figure 4. String Protein-Protein Interaction (PPI) enrichment p values of top ranked genes in the clinical/environmental datasets. Top 40 genes (~1% of *E. coli* genes) predicted by each method for ciprofloxacin and colistin clinical/environmental datasets were queried in Stringdb for PPI enrichment.

Dataset	Method	PPI enrichment p value
ciprofloxacin	EA-KS	5.82e-04
ciprofloxacin	EA-sum	0.0128
ciprofloxacin	Frequency	0.0254
colistin	EA-KS	3.25e-05
colistin	EA-sum	3.68e-05
colistin	Frequency	0.042

Additional References

1. Morais Cabral, J. H. *et al.* Crystal structure of the breakage-reunion domain of DNA gyrase. *Nature* **388**, 903–6 (1997).
2. Corbett, K. D., Schoeffler, A. J., Thomsen, N. D. & Berger, J. M. The structural basis for substrate specificity in DNA topoisomerase IV. *J. Mol. Biol.* **351**, 545–61 (2005).
3. Bellon, S. *et al.* Crystal structures of Escherichia coli topoisomerase IV ParE subunit (24 and 43 kilodaltons): a single residue dictates differences in novobiocin potency against topoisomerase IV and DNA gyrase. *Antimicrob. Agents Chemother.* **48**, 1856–64 (2004).
4. Kwon, H. J., Bennik, M. H., Demple, B. & Ellenberger, T. Crystal structure of the Escherichia coli Rob transcription factor in complex with DNA. *Nat. Struct. Biol.* **7**, 424–30 (2000).
5. Tomoike, F. *et al.* Indispensable residue for uridine binding in the uridine-cytidine kinase family. *Biochem. Biophys. reports* **11**, 93–98 (2017).
6. Lou, Y.-C. *et al.* Structure and dynamics of polymyxin-resistance-associated response regulator PmrA in complex with promoter DNA. *Nat. Commun.* **6**, 8838 (2015).
7. Han, X. *et al.* Crystal structures of ligand-bound octaprenyl pyrophosphate synthase from Escherichia coli reveal the catalytic and chain-length determining mechanisms. *Proteins* **83**, 37–45 (2015).
8. Wagih, O. *et al.* A resource of variant effect predictions of single nucleotide variants in model organisms. *Mol. Syst. Biol.* **14**, e8430 (2018).
9. Ng, P. C. & Henikoff, S. Predicting deleterious amino acid substitutions. *Genome Res.* **11**, 863–74 (2001).
