# Supplementary material for: Evolutionary action of mutations reveals antimicrobial resistance genes in Escherichia coli
Source: Nat Commun. 2022 Jun 9;13:3189. doi: 10.1038/s41467-022-30889-1 (PMC9184624; doi:10.1038/s41467-022-30889-1)
Supplement: Supplementary file 8 — Reporting Summary [file 41467_2022_30889_MOESM8_ESM.pdf]

nature portfolio

Corresponding author(s): David C. Marciano and Olivier Lichtarge

Last updated by author(s): Apr 18, 2022

Reporting Summary

Nature Portfolio wishes to improve the reproducibility of the work that we publish. This form provides structure for consistency and transparency in reporting. For further information on Nature Portfolio policies, see our Editorial Policies and the Editorial Policy Checklist.

Statistics

For all statistical analyses, confirm that the following items are present in the figure legend, table legend, main text, or Methods section.

n/a	Confirmed	
☐	☒	The exact sample size (n) for each experimental group/condition, given as a discrete number and unit of measurement
☐	☒	A statement on whether measurements were taken from distinct samples or whether the same sample was measured repeatedly
☐	☒	The statistical test(s) used AND whether they are one- or two-sided<br>*Only common tests should be described solely by name; describe more complex techniques in the Methods section.*
☒	☐	A description of all covariates tested
☐	☒	A description of any assumptions or corrections, such as tests of normality and adjustment for multiple comparisons
☐	☒	A full description of the statistical parameters including central tendency (e.g. means) or other basic estimates (e.g. regression coefficient) AND variation (e.g. standard deviation) or associated estimates of uncertainty (e.g. confidence intervals)
☐	☒	For null hypothesis testing, the test statistic (e.g. F, t, r) with confidence intervals, effect sizes, degrees of freedom and P value noted<br>*Give P values as exact values whenever suitable.*
☒	☐	For Bayesian analysis, information on the choice of priors and Markov chain Monte Carlo settings
☒	☐	For hierarchical and complex designs, identification of the appropriate level for tests and full reporting of outcomes
☐	☒	Estimates of effect sizes (e.g. Cohen's d, Pearson's r), indicating how they were calculated

Our web collection on statistics for biologists contains articles on many of the points above.

Software and code

Policy information about availability of computer code

Data collection	Sequencing reads were mapped to the E. coli K-12 MG1655 reference genome and variants were called by breseq version 0.31.0 using the "--no-junction-prediction" option to ignore reads that partially map to the reference and "-p" option to estimate allele frequency in the population.
Data analysis	An aggregate ranking algorithm (R package: RobustRankAggreg version 1.1) based on order statistics was used to combine three EA integral lists or frequency lists from the same antibiotic condition into one single list. The ET scores were color mapped to the protein structures with PyMOL 2.5.2 (Schrodinger, LLC). The code used in the analyses presented here can be viewed and accessed at https://github.com/LichtargeLab/EA_antibiotics_resistance.

For manuscripts utilizing custom algorithms or software that are central to the research but not yet described in published literature, software must be made available to editors and reviewers. We strongly encourage code deposition in a community repository (e.g. GitHub). See the Nature Portfolio guidelines for submitting code & software for further information.

Data

Policy information about availability of data

All manuscripts must include a data availability statement. This statement should provide the following information, where applicable:

- Accession codes, unique identifiers, or web links for publicly available datasets
- A description of any restrictions on data availability
- For clinical datasets or third party data, please ensure that the statement adheres to our policy

The raw sequencing data generated in this study have been deposited in the Sequence Read Archive database under SRA accession code PRJNA543834 https://www.ncbi.nlm.nih.gov/bioproject/PRJNA543834/. The E. coli K12 MG1655 reference sequence U00096.3 (https://www.ncbi.nlm.nih.gov/nuccore/U00096) was

used to map reads and make SNP calls. Protein Data Bank entries for gyrA (PDB: 1AB4 [http://doi.org/10.2210/pdb1AB4/pdb]), parC (PDB: 1ZVU [http://doi.org/10.2210/pdb1ZVU/pdb]), parE (PDB: 1S16 [http://doi.org/10.2210/pdb1S16/pdb]), rob (PDB: 1D5Y [http://doi.org/10.2210/pdb1D5Y/pdb]), udk (homolog from Thermus thermophilus, PDB: 3W8R [http://doi.org/10.2210/pdb3W8R/pdb]), basR (homolog from Klebsiella pneumoniae JM45, PDB: 4S04 [http://doi.org/10.2210/pdb4S04/pdb]) and ispB (PDB: 3WJK [http://doi.org/10.2210/pdb3WJK/pdb]) were used to map ET scores on structures. The data generated in this study are provided in the Supplementary Information/Source Data file. EA scores for all mutations in E. coli MG1655 is available on our website (http://bioheat.lichtargelab.org).

Field-specific reporting

Please select the one below that is the best fit for your research. If you are not sure, read the appropriate sections before making your selection.

- [x] Life sciences
- [ ] Behavioural & social sciences
- [ ] Ecological, evolutionary & environmental sciences

For a reference copy of the document with all sections, see nature.com/documents/nr-reporting-summary-flat.pdf

Life sciences study design

All studies must disclose on these points even when the disclosure is negative.

Sample size	An excess ALE of mutator clone samples were sequenced to ensure known positive control ciprofloxacin resistance drivers could be resolved from the background of mutated genes. Subsequent downsampling analysis indicated a 2-3 fold excess of samples were sequenced. Competition sample sizes were chosen based the ability to conveniently prepare DNA for sequencing. These samples sizes are sufficient because known positive controls are shown to significantly influence fitness in the assay.
Data exclusions	Sequencing reads which partially mapped to the reference sequence were ignored.
Replication	The ALE population replicates included E. coli MG1655 grown in the presence of ciprofloxacin (n=14), E. coli MG1655 grown in the presence of nucleotide analogs and ciprofloxacin (n=15), a highly mutagenic E. coli mutD5 ΔmutL::zeoR grown in the presence of ciprofloxacin (n=29), E. coli MG1655 grown in the presence of colistin (n=17), in the presence of nucleotide analogs and colistin (n=20) and E. coli mutD5 ΔmutL::zeoR grown in the presence of colistin (n=31). In the competition assays, n=5 independent biological samples for udk, rstB, and ispB were used. N=8 independent biological samples were used for rob and mutL. All other genes were repeated with n=6 independent biological samples. All attempts at replication were successful.
Randomization	In the competition assay, aliquots of cell mixtures are randomly assigned to antibiotics treatment group or no treatment group.
Blinding	Blinding is not performed in this study. Only cell-based and molecular biology experiments are performed. Assay results have quantifiable outputs (e.g. OD600, SNP calls, electropherogram results) that are less likely to be influenced by experimenter bias.

Reporting for specific materials, systems and methods

We require information from authors about some types of materials, experimental systems and methods used in many studies. Here, indicate whether each material, system or method listed is relevant to your study. If you are not sure if a list item applies to your research, read the appropriate section before selecting a response.

Materials & experimental systems

n/a	Involved in the study	
[x]	[ ]	Antibodies
[x]	[ ]	Eukaryotic cell lines
[x]	[ ]	Palaeontology and archaeology
[x]	[ ]	Animals and other organisms
[x]	[ ]	Human research participants
[x]	[ ]	Clinical data
[x]	[ ]	Dual use research of concern

Methods

n/a	Involved in the study	
[x]	[ ]	ChIP-seq
[x]	[ ]	Flow cytometry
[x]	[ ]	MRI-based neuroimaging
